# An optimized grapevine RNA isolation procedure and statistical determination of reference genes for real-time RT-PCR during berry development

**DOI:** 10.1186/1471-2229-6-27

**Published:** 2006-11-14

**Authors:** Karen E Reid, Niclas Olsson, James Schlosser, Fred Peng, Steven T Lund

**Affiliations:** 1 Faculty of Land and Food Systems, University of British Columbia, Vancouver, Canada

## Abstract

**Background:**

Accuracy in quantitative real-time RT-PCR is dependent on high quality RNA, consistent cDNA synthesis, and validated stable reference genes for data normalization. Reference genes used for normalization impact the results generated from expression studies and, hence, should be evaluated prior to use across samples and treatments. Few statistically validated reference genes have been reported in grapevine. Moreover, success in isolating high quality RNA from grapevine tissues is typically limiting due to low pH, and high polyphenolic and polysaccharide contents.

**Results:**

We describe optimization of an RNA isolation procedure that compensates for the low pH found in grape berries and improves the ability of the RNA to precipitate. This procedure was tested on pericarp and seed developmental series, as well as steady-state leaf, root, and flower tissues. Additionally, the expression stability of actin, AP47 (clathrin-associated protein), cyclophilin, EF1-α (elongation factor 1-α), GAPDH (glyceraldehyde 3-phosphate dehydrogenase), MDH (malate dehydrogenase), PP2A (protein phosphatase), SAND, TIP41, α-tubulin, β-tubulin, UBC (ubiquitin conjugating enzyme), UBQ-L40 (ubiquitin L40) and UBQ10 (polyubiquitin) were evaluated on *Vitis vinifera *cv. Cabernet Sauvignon pericarp using three different statistical approaches. Although several of the genes proved to be relatively stable, no single gene outperformed all other genes in each of the three evaluation methods tested. Furthermore, the effect of using one reference gene versus normalizing to the geometric mean of several genes is presented for the expression of an aquaporin and a sucrose transporter over a developmental series.

**Conclusion:**

In order to quantify relative transcript abundances accurately using real-time RT-PCR, we recommend that combinations of several genes be used for normalization in grape berry development studies. Our data support GAPDH, actin, EF1-α and SAND as the most relevant reference genes for this purpose.

## Background

Transcriptomics is an important growing field of molecular biology. Gene expression analyses are increasing our understanding of signalling and metabolic pathways underlying developmental and cellular processes. Real-time RT-PCR is currently one of the more powerful and sensitive techniques for analyzing gene expression. It provides outstanding accuracy of RNA quantification and has a broad dynamic range over wide experimental conditions [1-7]. As in other expression studies, data normalization is essential to control for experimental error introduced throughout sample preparation. It has been shown that real-time RT-PCR results are highly dependent on the reference genes chosen [8], which supports putting considerable effort into validating gene(s) chosen for normalization prior to extensive experimentation. Useful reference genes must not only be present in all samples but the expression levels need to remain constant relative to experimental pressures introduced. Data normalization can be problematic and several strategies have been reviewed [9].

Housekeeping genes are constitutively expressed and required for cellular survival, including functions such as cell wall structure and primary metabolism. Previously, these have been found to be reasonable internal reference genes for normalizing real-time data. These genes are expected to exhibit minor differences in their expression profiles under diverse experimental conditions. Examples such as GAPDH, 18S rRNA and EF1-α have been widely used in RNA blot analyses and are commonly used for real-time RT-PCR in various plant species [2,3,6,7,10,11]. While these genes have been found to be appropriate for some experiments, other candidates were recently reported to outperform these classical ones [12].

Grape berries undergo significant metabolic changes throughout their development, orchestrated in part by the up and down regulation of transcripts. This development follows a double sigmoidal pattern characterized by two periods of cellular expansion separated by a period of slowed growth [13]. The ability to identify transcripts that are resistant to growth fluctuations or stresses is challenging; therefore, it is important to identify candidate reference genes that are subject to only minimal regulation during an individual experiment, permitting accurate transcriptional analyses. To date, a limited number of real-time RT-PCR experiments focusing on grape berries has been published. Based on microarray and real-time RT-PCR data, UBQ-L40 [14] and one paralog of EF1-α [2] were previously reported as being stably expressed in grape berries.

Prior to evaluating expression patterns in biological samples, it is important to ensure that the RNA being used for cDNA synthesis is pure and not degraded. Grapevine tissues, like those in many higher plant species, contain abundant polyphenolic and polysaccharide compounds which cause challenges when isolating RNA. At full maturity, for example, Cabernet Sauvignon berries contain approximately 26 percent soluble solids, mainly glucose and fructose, and these sugars can co-precipitate with nucleic acids into a viscous gelatin-like pellet during RNA isolation. Moreover, due to the low RNA content in the maturing berries, success is limited in capturing low concentrations of nucleic acids using large-volume extraction protocols.

In this study, we present an RNA isolation protocol adapted from a previously described procedure developed for the evergreen tree, *Cinnamomum tenuipilum *[15]. Our protocol compensates for the acidic nature of grape berries and introduces modifications to both increase RNA yield and minimize contaminating polysaccharides. We demonstrate that high quality and quantity of RNA can be obtained from grape berries from all developmental stages as well as other grapevine tissues including flowers, leaves, and roots. Additionally, we present the expression patterns of 15 primer pairs targeting 14 commonly used reference genes that represent different functional classes in developing grape berries. Two different growing seasons were used in this study. Included are primer pairs that target either a single gene or two or more members of a gene family [5,10]. Three different statistical approaches were used to evaluate the reference genes; 1) cycle threshold (C_t_) range and coefficient of variance; 2) analysis using the geNorm software [16]; and 3) deviations from the C_t _mean [17]. Lastly, we demonstrate the effects of using one or more reference genes on the relative expression levels of an aquaporin and a sucrose transporter during grape development.

## Results and discussion

Microarray datasets can be a rich source of information for selecting real-time RT-PCR reference genes, as was done for Arabidopsis using the large public collection of data from Affymetrix GeneChip experiments [12]. Unfortunately, for emerging organisms like grapevine, public datasets are limited [2,18]. In this study, we set out to evaluate the stability of 15 primer pairs via three independent analytical methods to rank the effectiveness of internal reference genes for grape berries. Among these, 18S rRNA, GAPDH, actin and EF1-α are among the most commonly reported reference genes, but to date, no single candidate has been shown to be universally acceptable.

### Reference gene analysis

**Figure 1:**
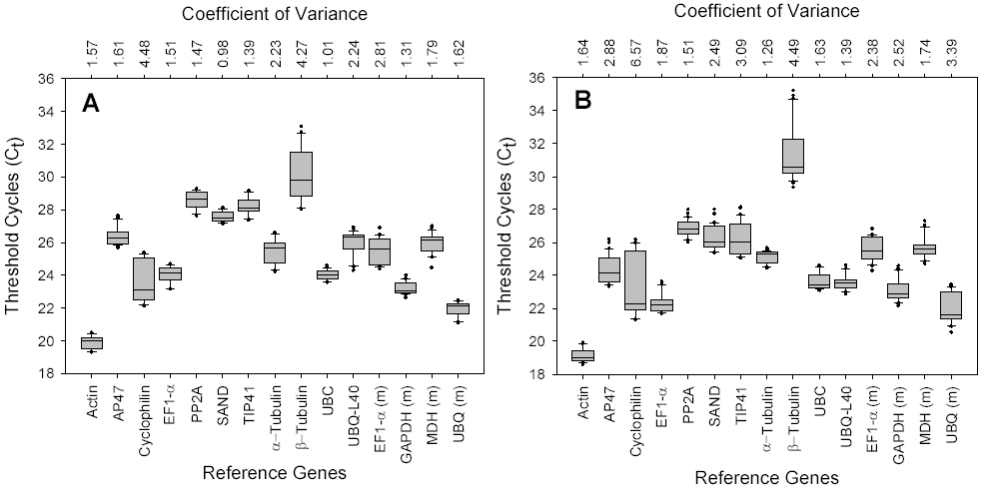
**Absolute C_t _from 2003 pericarp (A) and 2004 pericarp (B) samples**. Each box indicates 25/75 percentiles. Whisker caps represent 10/90 percentiles. The median is depicted by the line and all outliers are indicated by dots.

When assessing a set of reference genes, the evaluation method implemented can be a source of bias based on the assumptions underlying each approach. In an effort to minimize bias, the datasets were analysed using three different statistical approaches to identify the most stably expressed genes during grape berry development. The first, most straightforward method was to assess the C_t _range and calculate the coefficient of variance for each gene over two growing seasons. This allowed for a visual assessment of genes that had a narrow C_t _range over the entire developmental period. Naturally, the least amount of variance is most favourable. C_t _values for the 14 genes (15 primer pairs) ranged from 19 to 34, while the majority of these values were between 20 and 26 (Figure 1). Actin was the most abundant transcript, reaching threshold fluorescence after only 19 to 20 amplification cycles, whereas the C_t _average of all genes within the datasets was approximately 24 cycles. As a result, the actin transcript levels were around 32-fold more abundant than the dataset's average. The least abundant transcripts were β-tubulin, PP2A, and SAND, with C_t _values of 26 or higher. The calculated coefficient of variance of the C_t _values gives an indication of the expression stability of a particular gene. β-tubulin and cyclophilin each had large variances in their expression levels and the only CV values over 4.0 for both 2003 and 2004 pericarp sample sets. On the other hand, there was little consistency in genes with minimal CV. This method, although reasonable, did not clearly define any stably expressed reference genes across the developmental series in the two seasons. A disadvantage of this approach is that it overlooks RT-PCR variations between genes, samples, and to a lesser extent their repeats, meaning that experimental errors are likely present but not characterized.

**Figure 2:**
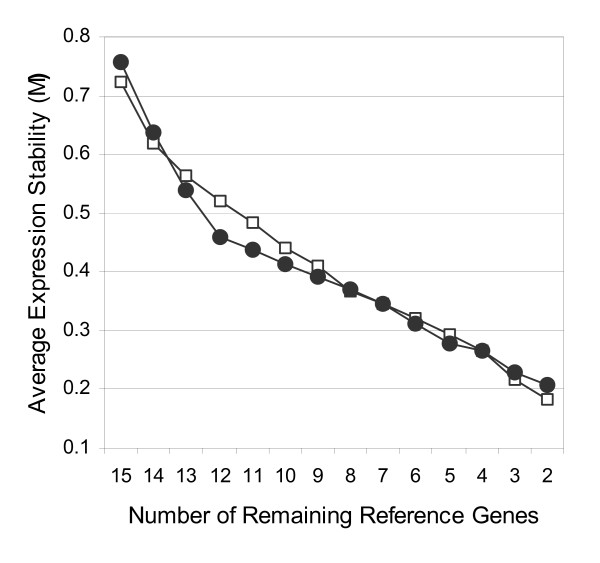
**geNorm ranking of reference genes over a grape berry development series**. 2003 pericarp series (□) and 2004 pericarp series (●). Ranked genes are shown in Table 2. Initially, expression stabilities (M) are calculated for each gene and the average M for all genes is plotted. Then the least stable gene is eliminated from the set and new M values are calculated in an iterative process until only two genes remain.

geNorm software was tested as a second means of assessing candidate reference genes [16]. This program calculates a gene expression stability measure (M) for each gene based on the average pairwise expression ratio between it and each of the other genes being studied. geNorm then performs a stepwise exclusion of the least stable gene and recalculates M until only two genes are left, these being the most stably expressed (Figure 2 and Table 2). Each gene studied had a relatively low M value in accordance with the limit of <1.5 suggested by geNorm. Only the β-tubulin surpassed this limit with an M value of 1.54 in the 2004 pericarp dataset. GAPDH (m) ranked among the top three genes in both the 2003 and 2004 sample sets. Cyclophilin, β-tubulin and EF1-α (m) consistently ranked poorly, indicating that these genes are not stably expressed and likely play a functional role in one or more stages of berry development. These findings are supported by reports describing differential expression of cyclophilin [18] and β-tubulin [2] in microarray experiments. Next, pairwise variation is calculated by geNorm to determine the fewest number of reference genes necessary for accurate normalization. As suggested [16], pairwise variation values below 0.15 do not require greater than two control genes. Likewise, in our experiment where the pairwise variation was less than 0.15 (data not shown), geometric averaging of only the top two genes would be needed for accurate normalization, when examining our datasets. The geNorm algorithm is dependent on the assumption that none of the genes being analyzed are co-regulated; otherwise, these genes would be inaccurately selected as favourable candidates. In this experiment, EF1-α (m) and EF1-α should benefit from a pairwise comparison program like geNorm when included in the same dataset. We found, however, that irrespective of whether EF1-α (m) was included in the dataset with EF1-α or not, geNorm consistently ranked EF1-α (m) poorly.

The third statistical approach tested is based on the idea that the mean expression of candidate reference genes (mean C_t_) reflects the most optimal normalization, assuming that all genes have independent cellular functions [17]. The calculated mean difference in expression reflects the constant difference in expression levels between a gene and the mean of the dataset (C_t _- mean C_t_). For example, a C_t _of -5.7 for actin indicates that it took fewer PCR cycles to reach the mean C_t_, whereas a C_t _of 2.7 for SAND indicates that more cycles were needed (Table 3). In our experiment, cyclophilin, β-tubulin, and EF1-α (m) were removed from the analysis based on the conclusions drawn from the previous two methods that these were not stably expressed and would otherwise artificially skew the mean C_t_. Previously, Brunner *et al*. [10] showed that two of their most stable reference genes represented both high and low expression levels compared to the genes being analysed, demonstrating that the level of expression of the reference genes does not affect accuracy. In our study, reference genes were ranked based on their deviation from the mean (2× standard deviation) (Table 3). Highest ranked were SAND and actin in 2003 and 2004 pericarp, respectively, with minimal deviation around the mean of all other reference genes in the dataset.

In addition to primer pairs that target single transcripts, we set out to evaluate a subset of our primer pairs that should amplify paralogous transcripts, based on comparative sequence analyses [5]. Primer pairs MDH (m), GAPDH (m), UBQ (m) and EF1-α (m) each targeted conserved sequences in a minimum of two known transcripts within their gene families (Table 1). Our results demonstrated no consistent trend in effectiveness of targeting multiple transcripts; while GAPDH (m) consistently ranked relatively high in all analyses, EF1-α (m) fared poorly in all three approaches.

The most prominent observation after completing the three analysis methods was that each produced a different set of top ranked candidates. Furthermore, these results were not consistent between sampling years. Generally, we found that the top ranking reference genes remained high on the lists in each analysis method, but given that each method resulted in different units, we recognized the need to devise a scheme to standardize the units while maintaining the distribution of results. CV, M, and 2× SD results were each given a scaled value based on the distribution within each dataset (2003 and 2004, independently). Cyclophilin, β-tubulin, and EF1-α (m) were excluded since they were not included in all of the evaluation methods. When 2003 and 2004 dataset scores were combined, GAPDH (m) ranked highest across all reference genes, followed by actin, EF1-α, and SAND.

Due to seasonal differences observed using the different approaches (Figure 1; Tables 2 and 3), further validation was initiated to test whether the top candidates continued to perform well in additional berry samples. A second 2003 sample was generated using the mesocarp tissue (berry flesh without skin). Expression studies were performed in the same manner as was done earlier with the pericarp tissue. The top four gene candidates in the 2003 mesocarp tissue, based on the same three statistical approaches, were actin, GAPDH (m), TIP41 and UBC, but once all datasets (2003 and 2004 pericarp, and 2003 mesocarp) were tabulated, the top ranked genes were GAPDH (m), actin, EF1-α and SAND (data not shown). These data demonstrate that if subtle changes in expression are of critical importance, choosing a single reference gene may not be universally suitable.

### Reference gene validation

**Figure 3:**
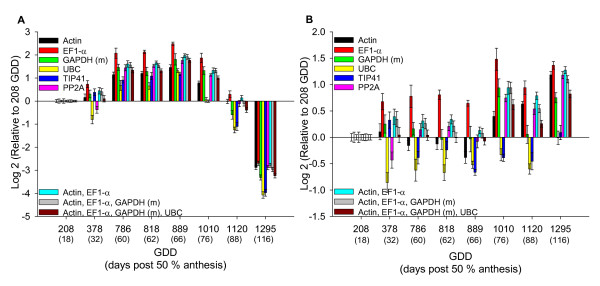
**Expression levels of an aquaporin (A) and a sucrose transporter (B) during berry development**. Genes were normalized to individual and/or combined reference genes. Developmental stages are defined by growing degree days (GDD), which are calculated by taking the average of the daily high and low temperature each day compared to a baseline (10°C). Data points are compared relative to the first sampling stage, 208 GDD. During the 2004 season, veraison, which is the period of berry ripening initiation, occurred between 786 and 889 GDD.

The use of one or multiple reference gene(s) in the calculation of relative expression data can have a significant influence on the final normalized results. To test the effect of reference gene selection on the outcome of a practical experiment, we evaluated the relative expression patterns for two functionally unrelated genes, an aquaporin [19] and a sucrose transporter [20], using different reference genes (Figure 3). An ideal result would have been for both genes to have had consistent expression patterns, irrespective of the reference gene used for normalization. This was the case for the aquaporin (Figure 3A); this expression pattern was consistent with that reported by Picaud *et al*. [19], who found lower expression levels in earlier stages of berry development. Conversely, relative transcript abundance patterns for the sucrose transporter were dependent on the gene(s) used for normalization (Figure 3B). The inconsistent sucrose transporter profiles demonstrated that using only one gene for normalization can lead to over or under estimation of relative transcript abundance. Davies *et al*. [20] used RNA blot analyses to evaluate the expression pattern of the sucrose transporter, VvSUC12 [GenBank: AF021809] in Shiraz berries and showed a down regulation from fruit set through to veraison (ripening initiation, when the berries change from green to red), then a continued up regulation through to maturity. In this study, our sucrose transporter data were consistent with those of Davies *et al*. [20] when actin, UBC, and the combination of actin, EF1-α and GAPDH (m) were used for normalization. Other reference genes, when used independently for normalization, led to different conclusions regarding relative expression levels throughout berry development (Figure 3B). Once again, these results reinforce the importance of validating reference gene(s) prior to experimental applications, and more importantly, taking the geometric mean of a greater number of genes for normalization.

### RNA isolation

**Figure 4:**
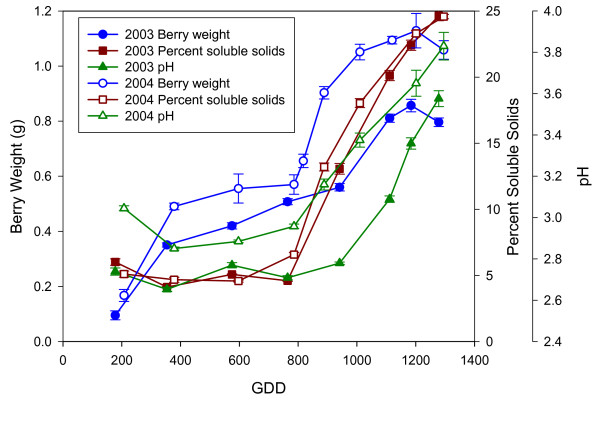
**Cabernet Sauvignon berry development through the 2003 and 2004 seasons**. Berry weight, soluble solids, and pH values were generated from 4 replicates of 50 berries, taken from 3 clusters each. Soluble solids were determined using a digital refractometer (PR-101, Atago, Japan) and the pH was measured on a pH meter (PHM82, Bach Simpson Ltd., London, Canada).

**Figure 5:**
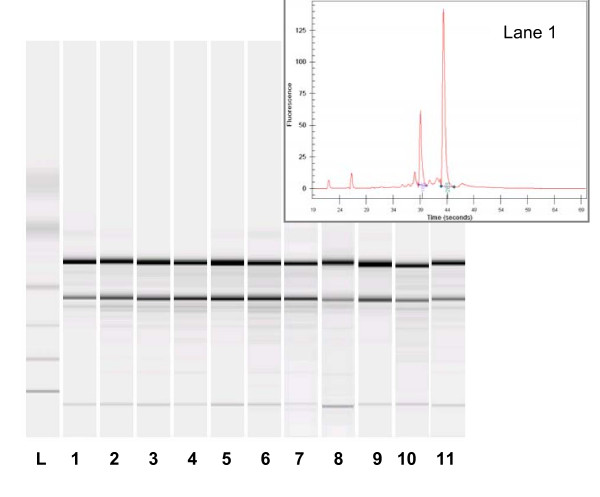
**Total RNA samples run on an Agilent 2100 Bioanalyzer RNA 6000 Nano LabChip**. L, Ladder/marker; 1–2, pre-veraison pericarp; 3, veraison pericarp; 4, post-veraison pericarp; 5–6, pre-veraison seed; 7, veraison seed; 8, post-veraison seed; 9, root; 10, pre-anthesis flower; 11, post-anthesis flower. The fluorescence plot shown in the upper right corner is an example of the electropherogram with 18S and 28S peaks.

Young grape berries have a pH between 2.0 and 3.0 (Figure 4), while ripe berries contain high levels of polysaccharide compounds (percent soluble solids, shown in Figure 4) and polyphenolics. These characteristics have made it challenging to extract quality RNA from these and other grapevine tissues [21,22]. Development of a single robust RNA isolation protocol for all grapevine tissues can minimize technical variation when comparing real-time RT-PCR experiments or other downstream applications such as microarray analyses. We present in Methods, adaptations made to a protocol originally published for the woody plant, *C. tenuipilum *[15], modified to accommodate a variety of grapevine tissues. In this study, total RNA was extracted from grape pericarp, seeds, leaves, roots and flowers. Analyses of total RNA extracts demonstrated that they were each of high quality (Figure 5). This is the first report of a robust RNA isolation protocol applicable to diverse grapevine tissues. Our modifications account for the acidic nature of grape berries by increasing the buffering capacity from 100 mM to 300 mM Tris HCl in the extraction buffer, resulting in increased yields in early-staged berries. As well, an alcohol precipitation step was included to concentrate the RNA in solution prior to selectively precipitating the RNA with LiCl. Increased yields were achieved when the RNA was concentrated just prior to the addition of LiCl. This was especially true for mature pericarp and seeds when cellular activity has diminished and the water content in the pericarp has peaked. Yields were quite diverse depending on the source of the tissue. Leaves yielded the highest amount of total RNA (400–600 μg/gfw), followed by flowers, roots and pre-veraison seeds (150–300 μg/gfw), pre-veraison pericarp (40–120 μg/gfw), post-veraison pericarp (15–30 μg/gfw) and the lowest being from post-veraison seeds (3–10 μg/gfw). The differences observed were likely related to the developmental and metabolic properties of each distinct tissue.

## Conclusion

Our findings support that reference gene selection has a significant effect on normalized gene expression data in real-time RT-PCR experiments. We demonstrated that the most stable reference genes ranked among the top genes when data from three independent statistical approaches were evaluated but no single gene was consistently best. For more accurate normalization, use of at least two of the top ranked reference genes followed by geometric averaging is recommended for determining a normalization factor. Specifically for grape berries, GAPDH (m) used together with actin, a previously reported primer set for a specific EF1-α [2], or SAND are most stable for grape berry development studies. Our conclusion is supported by data representing two growing seasons.

## Methods

### Plant material

Pre- and post-anthesis flowers, berries, leaves and roots (produced through air-layering) from *Vitis vinifera *cv. Cabernet Sauvignon (clone 15 grafted on rootstock 101-14) were collected from vines located in Osoyoos, British Columbia, Canada, during the 2003 and 2004 field seasons. All tissues were immediately frozen in liquid nitrogen upon collection and then stored at -80°C. Seeds were removed from the berries by gently breaking open the berries under liquid nitrogen, then pericarp (skin and flesh) and seed portions were stored separately until further use. During the 2003 season, eight samples were collected to reflect the entire development series, whereas in 2004 an attempt was made to capture more veraison stages (the onset of ripening), so ten samples were used.

### Total RNA isolation

The extraction buffer contained 300 mM Tris HCl (pH 8.0), 25 mM EDTA, 2 M NaCl, 2% CTAB, 2% PVPP, 0.05% spermidine trihydrochloride, and just prior to use, 2% β-mercaptoethanol. Tissue was ground to a fine powder in liquid nitrogen using a mortar and pestle. The powder was added to pre-warmed (65°C) extraction buffer at 20 ml/g of tissue and shaken vigorously. Since berries have higher water content than other grape tissues, a lower extraction buffer ratio of 10–15 ml/g weight was sufficient. Tubes were subsequently incubated in a 65°C water bath for 10 min and shaken every couple of min. Mixtures were extracted twice with equal volumes chloroform:isoamyl alcohol (24:1) then centrifuged at 3,500 × g for 15 min at 4°C. The aqueous layer was transferred to a new tube and centrifuged at 30,000 × g for 20 min at 4°C to remove any remaining insoluble material. This step proved more critical for root and flower tissues. To the supernatant, 0.1 vol 3 M NaOAc (pH 5.2) and 0.6 vol isopropanol were added, mixed, and then stored at -80°C for 30 min. Nucleic acid pellets (including any remaining carbohydrates) were collected by centrifugation at 3,500 × g for 30 min at 4°C. The pellet was dissolved in 1 ml TE (pH 7.5) and transferred to a microcentrifuge tube. To selectively precipitate the RNA, 0.3 vol of 8 M LiCl was added and the sample was stored overnight at 4°C. RNA was pelleted by centrifugation at 20,000 × g for 30 min at 4°C then washed with ice cold 70% EtOH, air dried, and dissolved in 50–150 μl DEPC-treated water.

### RNA purification and cDNA synthesis

RNA concentration and 260/280 nm ratios were determined before and after DNase I digestion with a NanoDrop ND-1000 spectrophotometer (NanoDrop Technologies, Wilmingon, DE, USA), and 1% agarose gels were run to visualize the integrity of the RNA. To improve our ability to visually assess RNA quality, the same RNA samples were run on an Agilent 2100 Bioanalyzer RNA 6000 Nano LabChip (Agilent, Mississauga, ON, Canada), as shown in Figure 5. Total RNA was purified using an RNeasy kit (Qiagen, Valencia, CA, USA) with the addition of an on-column DNase I digestion. cDNAs were synthesized from 2 μg of total RNA using the Superscript III first-strand synthesis system followed by the RNase H step (Invitrogen, Carlsbad, CA, USA), according to the manufacturer's instructions.

### PCR primer design

Several common housekeeping genes were selected for expression analysis (Table 1). Primers were designed using Primer Express 2.0 software (Applied Biosystems, Foster City, CA, USA) with melting temperatures (Tm) of 58–60°C, primer lengths of 20–24 bp, and amplicon lengths of 61–150 bp. The majority of the primer pairs targeted a single gene within a given gene family with the exceptions of MDH (m), GAPDH (m), UBQ (m) and EF1-α (m) primer pairs (m, representing multiple gene family members targeted).

### Real-time PCR conditions and analysis

PCR reactions were performed in 96-well plates with an ABI PRISM^® ^7500 Sequence Detection System (Applied Biosystems) using SYBR^® ^Green to detect dsDNA synthesis. Reactions were done in 25 μl volumes containing 200 nM of each primer, 5 μl cDNA (corresponding to ~3 ng), and 12.5 μl 2× SYBR Green Master Mix Reagent (Applied Biosystems). Aliquots from the same cDNA sample were used with all primer sets in each experiment. Reactions were run using the manufacturer's recommended cycling parameters of 50°C for 2 min, 95°C for 10 min, 40 cycles of 95°C for 15 s, and 60°C for 1 min. No-template controls were included for each primer pair and each PCR reaction was completed in triplicate. Dissociation curves for each amplicon were then analyzed to verify the specificity of each amplification reaction; the dissociation curve was obtained by heating the amplicon from 60°C to 95°C (See additional file 1: Dissociation curve data).

Data were analyzed using the SDS 1.2.2 software (Applied Biosystems). Expression levels were determined as the number of amplification cycles needed to reach a fixed threshold in the exponential phase of the PCR reaction (C_t_). All amplification plots were analyzed with an Rn threshold of 0.2 to obtain C_t _values. The PCR efficiency was determined for each gene with LinReg software, which uses absolute fluorescence data captured during the exponential phase of amplification of each reaction [23]. Results from the SDS and LinReg software were imported into Microsoft Excel for further analyses and to correct for the different PCR efficiencies [24]. All primer pairs had efficiencies higher than 1.80 with the exception of EF1-α (m) (1.70) and β-tubulin (1.54). Each was run on the full pericarp developmental series for 2003 and 2004. In addition, all primer pairs except those targeting β-tubulin and cyclophilin were run on 2003 mesocarp samples.

In order to evaluate reference gene stability among samples, three statistical approaches were incorporated. In the first approach, C_t _difference (C_t max_-C_t min_) and CV were calculated for each gene throughout each development series tested. During the second approach, C_t _values were converted into relative quantities and imported into geNorm v.3.4 software [16]. Analyses were performed both with and without EF1-α (m) data to evaluate whether co-regulation with EF1-α biased the results, considering that the EF1-α (m) targets EF1-α as well as other paralogs. Finally, in the third approach, the standard deviation was calculated for each mean C_t _difference (C_t_-mean Ct) [17]. β-tubulin, cyclophilin, and EF1-α (m) were excluded from this third approach due to their poor performances during earlier analysis.

To score reference genes based on gene stability, a scoring scheme was implemented whereby results were combined from each statistical approach for each sampling year. Given that cyclophilin, β-tubulin and EF1-α (m) were excluded from the final analyses (Table 3), they could not be included in the final scoring scheme. For all other genes, the results from each statistical approach (CV, M or 2× SD) within a dataset (2003 or 2004 pericarp development series) were distributed and assigned a score value between 1 to 100, an arbitrary scoring range. For example, the CV values for the 2003 samples ranged from 0.98 for SAND to 2.24 for UBQ-L40. SAND was assigned an arbitrary value of 1 and UBQ-L40, an arbitrary value of 100. Then all other genes were assigned values between 1 and 100, scaled based on their relative distribution. Once all scores were derived for each statistical approach, a cumulative score was used to deduce the final standing.

## Abbreviations

CTAB, cetyltrimethylammonium bromide; DEPC, diethyl pyrocarbonate; GDD, growing degree days; gfw, gram fresh weight.

## Authors' contributions

KER developed the RNA extraction protocol, assisted in real-time data analyses including devising the scoring system to make data comparisons amongst the reference gene evaluation methods, and drafted the manuscript. NO designed and conducted the real-time RT-PCR experiments, conducted real-time data analyses, and assisted in drafting the manuscript. JS was responsible for coordinating and carrying out grape tissue collections from the commercial vineyard. FP assisted NO with DNA template selection and real-time primer design. STL supervised the study and edited the manuscript, which was approved by all authors.

## Supplementary Material

Additional file 1 Dissociation curve data. Click here for file

## Figures and Tables

**Table 1:** Genes and primer sets used for real time RT-PCR

*Gene abbreviation*	*GenBank Accession*	*Arabidopsis ortholog locus*	*Arabidopsis locus description*	*Primer pair (forward/reverse)*	Product size bp/efficiency*
Actin	EC969944	AT5G09810.1	Actin 7 (ACT7)/actin 2 (other hits include ACT1, ACT3, ACT4, ACT8, ACT11, ACT12)	CTTGCATCCCTCAGCACCTT/TCCTGTGGACAATGGATGGA	82/2.00
AP47	EC951857	AT5G46630.1	Clathrin adaptor complexes medium subunit family protein	GGTTCCCATGTTTACAGCATCTG/GCACCCACTCAACGGTATTGTAC	86/1.93
Aquaporin	EC969993	AT2G37170.1	Plasma membrane intrinsic protein 2B (PIP2B)/aquaporin PIP2.2 (PIP2.2)	TCCGCCAAGGACTATCATGAC/CGCAATCAGAGCCCTGTAGAA	90/1.93
Cyclophilin	EC969926	AT4G34870.1	Peptidyl-prolyl cis-trans isomerase/cyclophilin (CYP1)	GGAGCCTGAGCCTACCTTCTC/GTGTTCGGCCAGGTGGTAGA	66/1.86
EF1-α ^†^	EC959059	AT5G60390.1	Elongation factor 1-alpha (other hits include AT1G07940.2, AT1G07940.1, AT1G07920.1, AT1G07930.1)	GAACTGGGTGCTTGATAGGC/AACCAAAATATCCGGAGTAAAAGA	150/1.87
PP2A	CB980232	AT3G25800.1	Serine/threonine protein phosphatase 2A (PP2A) 65 KDa regulatory subunit A	TCGTGATGCTGCTGCTAACAA/TTGCCCAGTCAGGACCAAAT	62/1.90
SAND	CF405409	AT2G28390.1	SAND family protein	CAACATCCTTTACCCATTGACAGA/GCATTTGATCCACTTGCAGATAAG	76/1.91
Sucrose transporter	EC920891	AT2G02860.1	Sucrose transporter/sucrose-proton symporter	GGATAACTTCCCTGCCTCAATGA/TTCTTGTAGCAGCTGAGAGGATCA	67/1.91
TIP41	EC947050	AT4G34270.1	TIP41-like family protein	CATGCGAGTGTCCCTCAATCT/TCTCTTGCGTTTCTGGCTTAGA	61/1.92
α-Tubulin	EC930869	AT5G19780.1	Tubulin alpha-3/alpha-5 chain (TUA5) (other hits include TUA1-4, TUA6)	CAGCCAGATCTTCACGAGCTT/GTTCTCGCGCATTGACCATA	119/1.82
β-Tubulin	EC922104	AT1G75780.1	Tubulin beta-1 chain (TUB1)	TGAACCACTTGATCTCTGCGACTA/CAGCTTGCGGAGGTCTGAGT	86/1.54
UBC	EC922622	AT1G64230.1	Ubiquitin-conjugating enzyme	GAGGGTCGTCAGGATTTGGA/GCCCTGCACTTACCATCTTTAAG	75/1.92
UBQ-L40	EC929411	AT3G52590.1	Ubiquitin extension protein 1 (UBQ1)/60S ribosomal protein L40 (RPL40B)	CATAACATTTGCGGCAGATCA/TGGTGGTATTATTGAGCCATCCTT	80/1.89
EF1-α (m)	CB977561	AT5G60390.1	Elongation factor 1-alpha (other hits include AT1G07940.2, AT1G07940.1, AT1G07920.1, AT1G07930.1, AT5G60390.2)	CGCCTGTCAATCTTGGTCAGTAT/AATGGCTATGCCCCTGTTCTG	83/1.70
	CB975242				
	EC958777				
	EC931777				
	EC925368				
GAPDH (m)	CB973647	AT1G13440.1	Glyceraldehyde 3-phosphate dehydrogenase, cytosolic.	TTCTCGTTGAGGGCTATTCCA/CCACAGACTTCATCGGTGACA	70/1.84
	CB970967				
	CF515110				
	EC930334				
MDH (m) ^‡^	EC921711	AT5G43330.1	Malate dehydrogenase, cytosolic (other hit include AT1G04410.1 MDH cytosolic)	CCATGCATCATCACCCACAA/GTCAACCATGCTACTGTCAAAACC	72/1.85
	EC923897				
	EC921960				
	EC919995				
UBQ10 (m)	CB915250	AT4G02890.1	Polyubiquitin (UBQ14) (other hits include UBQ4, UBQ10, UBQ11)	CAAATGGCTGAGACCCACAA/TATCCCAGTGGTCGGTTGGT	73/1.88
	CB977307				

All grape sequences were named based on similarity to Arabidopsis proteins determined via BLASTX. In most cases, the name indicates only a gene family or subfamily rather than a specific member since partial grape sequences and BLAST searches do not necessarily identify the putative Arabidopsis ortholog. Closest Arabidopsis homologs were identified using TAIR BLAST 2.2.8.

* The PCR efficiency was determined with LinReg software [23].

^† ^Terrier *et. al*. [2].

^‡ ^(m) indicates that the primer pair was designed to target more than one gene family member.

**Table 2:** Reference genes ranked in descending order with respect to expression stability (M)

*geNorm ranking*	*2003 Pericarp*	*2004 Pericarp*
1 + 2	TIP41 + AP47	Actin + EF1-α
3	GAPDH (m)	GAPDH (m)
4	MDH (m)	UBQ-L40
5	SAND	MDH (m)
6	α-Tubulin	PP2A
7	EF1-α	UBC
8	Actin	α-Tubulin
9	UBQ-L40	SAND
10	PP2A	AP47
11	UBC	UBQ (m)
12	UBQ (m)	TIP41
13	EF1-α (m)	EF1-α (m)
14	β-Tubulin	Cyclophilin
15	Cyclophilin	β-Tubulin

**Table 3:** Mean expression difference and 2× SD between each single gene compared to the mean expression of the other 11 listed genes.

*2003 Pericarp*			*2004 Pericarp*		
Ranked genes*	Mean difference (C_t_)*	*Accuracy (2× SD)^†^*	*Ranked genes*	*Mean difference (C_t_)*	*Accuracy (2× SD)*

SAND	2.70	0.343	Actin	-5.33	0.462
GAPDH (m)	-2.10	0.504	PP2A	3.10	0.496
TIP41	3.38	0.600	EF1-α	-1.86	0.604
MDH (m)	1.00	0.638	SAND	2.52	0.626
EF1-α	-1.15	0.647	GAPDH (m)	-1.02	0.630
AP47	1.37	0.719	UBC	-0.43	0.689
Actin	-5.70	0.733	AP47	0.33	0.728
PP2A	3.79	1.015	UBQ-L40	-0.49	0.889
α-Tubulin	0.39	1.039	α-Tubulin	1.23	0.892
UBC	-1.19	1.202	MDH (m)	1.82	0.925
UBQ-L40	0.96	1.213	UBQ (m)	-2.23	0.984
UBQ (m)	-3.44	1.421	TIP41	2.36	1.057

Cyclophilin, EF1-α (m) and β-tubulin were excluded from analysis due to their low performances using geNorm.

* Mean difference (C_t_-mean C_t_).

^† ^Precision around the mean C_t _is represented by 2× the standard deviation.
