# Supplementary material for: An optimized grapevine RNA isolation procedure and statistical determination of reference genes for real-time RT-PCR during berry development
Source: BMC Plant Biol. 2006 Nov 14;6:27. doi: 10.1186/1471-2229-6-27 (PMC1654153; doi:10.1186/1471-2229-6-27)

Additional File 1.

Dissociation curves for PCR products amplified with the primer pairs listed in Table 1. NTC: no template control.

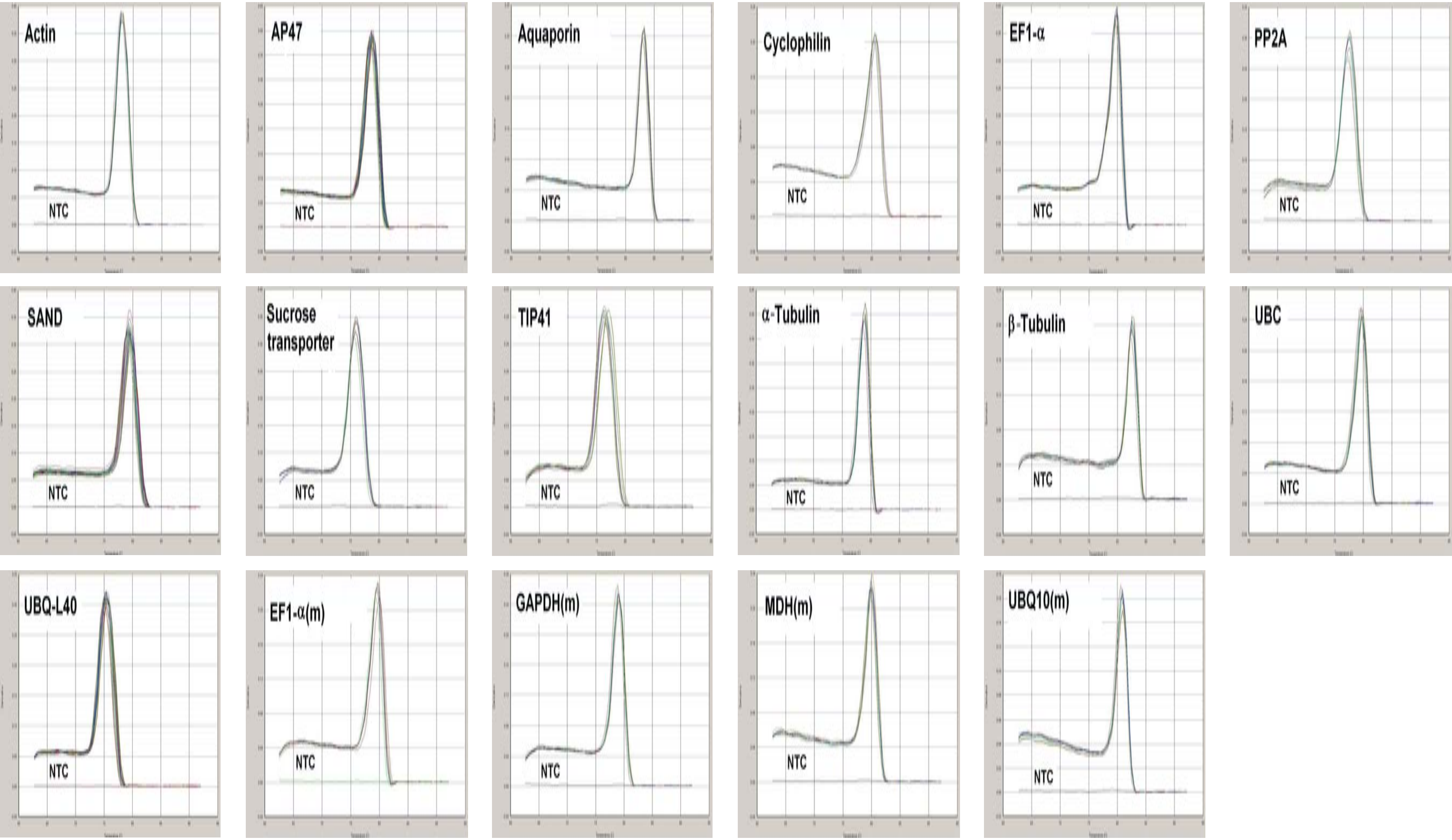

Supplement: Additional file 1 — Dissociation curve data. [file 1471-2229-6-27-S1.pdf]
